# Intraoperative application of mixed and augmented reality for digital surgery: a systematic review of ethical issues

**DOI:** 10.3389/fsurg.2024.1287218

**Published:** 2024-03-14

**Authors:** Frank Ursin, Cristian Timmermann, Lasse Benzinger, Sabine Salloch, Fabian-Alexander Tietze

**Affiliations:** ^1^ Institute for Ethics, History and Philosophy of Medicine, Hannover Medical School (MHH), Hannover, Germany; ^2^ Institute for Ethics and History of Health in Society, Medical Faculty, University of Augsburg, Augsburg, Germany; ^3^ Department of Psychiatry and Psychotherapy, Berlin Jewish Hospital, Academic Teaching Hospital of the Charité, Berlin, Germany

**Keywords:** augmented reality, mixed reality, extended reality, digital health, ethics

## Abstract

**Introduction:**

Head-mounted displays (HMDs) that superimpose holograms onto patients are of particular surgical interest as they are believed to dramatically change surgical procedures by including safety warning and allowing real-time offsite consultations. Although there are promising benefits of mixed and augmented reality (MR/AR) technologies in surgery, they also raise new ethical concerns. The aim of this systematic review is to determine the full spectrum of ethical issues that is raised for surgeons in the intraoperative application of MR/AR technology.

**Methods:**

Five bibliographic databases were searched for publications on the use of MR/AR, HMDs and other devices, their intraoperative application in surgery, and ethical issues. We applied qualitative content analysis to the *n* = 50 articles included. Firstly, we coded the material with deductive categories derived from ethical frameworks for surgical innovations, complications and research. Secondly, clinical aspects with ethical relevance were inductively coded as ethical issues within the main categories. Thirdly, we pooled the ethical issues into themes and sub-themes. We report our findings according to the reporting guideline RESERVE.

**Results:**

We found *n* = 143 ethical issues across ten main themes, namely patient-physician relationship, informed consent, professionalism, research and innovation, legal and regulatory issues, functioning equipment and optimal operating conditions, allocation of resources, minimizing harm, good communication skills and the ability to exercise sound judgement. The five most prevalent ethical issues are “Need for continuous research and innovation”, “Ensuring improvement of the learning curve”, “MR/AR enables new maneuvers for surgeons”, “Ensuring improvement of comfort, ergonomics, and usability of devices,” and “Not withholding MR/AR if it performs better”.

**Conclusions:**

Recognizing the evidence-based limitations of the intraoperative MR/AR application is of paramount importance to avoid ethical issues, but clinical trials in surgery pose particular ethical risks for patients. Regarding the digital surgeon, long-term impact on human workforce, potentially harmful “negative training,” i.e., acquiring inappropriate behaviors, and the fear of surveillance need further attention. MR/AR technologies offer not only challenges but significant advantages, promoting a more equitable distribution of surgical expertise and optimizing healthcare. Aligned with the core principle of social justice, these technologies enable surgeons to collaborate globally, improving training conditions and addressing enduring global healthcare inequalities.

## 1 Introduction

Extended reality (XR) technologies promise to provide important support for surgical interventions in patient education, preoperative planning and surgical navigation (1, 2). This technology belongs to the promising trend of “digital surgery,” i.e., the processing and visualization of surgical data sets for precision surgery (3), or “the use of technology for the enhancement of preoperative planning, surgical performance, therapeutic support, or training, to improve outcomes and reduce harm” (4). Augmented reality (AR) can provide important information, such as the monitoring of vital signs, clinical records, video calls to colleagues, operative instructions and checklists (see Box 1). Furthermore, mixed reality (MR) with three-dimensional (3D) hologram pictures of the operating area can enhance the spatial awareness of surgeons and enable geographically separated teams to jointly participate in an intervention requiring multiple expertise (5). MR promises to solve the important challenge that two-dimensional (2D) images taken preoperatively form the basis for 3D real-world surgical procedures. The hardware devices can be casual monitors, tablets, microscopes, exoscopes, endoscopes, head-up displays or head-mounted displays (HMD). The next logical step is to incorporate this technology into robotic surgical systems using minimally invasive surgical approaches as well.

Box 1 **Concepts and basic principles of the three types of extended reality (XR): augmented reality (AR), mixed reality (MR) and virtual reality (VR).**

Surgeons can receive additional information in their field of vision by wearing head-mounted displays that can superimpose virtual models on the real world. Extended reality (XR) is an umbrella term for augmented reality (AR), mixed reality (MR), and virtual reality (VR) (25). Not all HMDs can superimpose 3D holograms. Both AR and MR overlay virtual objects on the real world, but only MR provides the depth and perspective of the virtual elements, which influences the 3D visualization of the patient's anatomy during surgical procedures (26). While the natural surroundings are still visible with AR and MR, this is not the case with VR that virtually displaces a person to another (virtual) location (25). Virtual reality is better suited for surgical training and preoperative planning (20).

Two key technologies are necessary for utilizing MR/AR intraoperatively (27):

1. Data preparation for the visualization of medical imaging: 2D or 3D radiologic images taken preoperatively have to be computed into a 3D model by using image processing software to segment the anatomical region of interest, generate a 3D triangular surface model and, finally, plan the operation with surgical planning software.
2. Registration and tracking: “Registration is the process in which the visualized computer-generated object […] is superimposed and oriented into situs in the correct position.” (27) Tracking ensures that the visualization stays in the right position when moving in the 3D space.

Head-mounted displays are of particular surgical interest as they are believed to dramatically change surgical standards and techniques in preoperative planning, patient education and the operating theatre (6, 7). Those intraoperative applications especially offering image guidance and data display (8) are currently being explored by many surgical specialties such as urology (9), ophthalmology (10), neurosurgery (11), and visceral (12), vascular (13) and spinal surgery (14). The corresponding software allows for reconstructing 3D models that surgeons can then view on a stereoscopic HMD (15), surgical planning, telemedicine and patient education, and can stream ultrasound, laparoscopy and endoscopy images inside an MR view (16), as well as stereoelectroencephalographic interventions in patients with epilepsy, cerebral or kidney aneurysms, skull base tumors, cervical fractures and craniomaxillofacial surgery (17). Furthermore, using MR/AR HMDs in video-assisted surgery can mitigate ergonomic disadvantages of conventional monitors, such as limited freedom of movement resulting in muscular fatigue of the upper body (18).

Although the intraoperative use of MR/AR technology is in its early stages of development and implementation, proponents of the technology state that it is safe and improves surgical and health-economic outcomes (19, 20). However, only focusing on the post-operative outcomes neglects pre- and intraoperative data collection and processing (4). Although there are promising benefits of MR/AR in surgery, it also raises important ethical concerns. Similar to many medical technologies that reduce direct contact, medico-ethical issues include the datafication of patients and their conditions and the progressive erosion of the patient-physician relationship, which can lead to a reduction of empathy and loss of confidence in medical treatment. Although there is some work on the ethicality of MR/AR technologies in several branches (21–23), ethical issues of intraoperative MR/AR application in surgery are underexplored and have not yet been analyzed systematically (20).

Although a recent Delphi study with 38 experts covered ethical issues of digital surgery regarding artificial intelligence and patient data, MR/AR technology was not addressed specifically and systematically (4). The results of the Delphi study regarding ethical issues, namely on privacy, confidentiality, public trust, and consent, only cover a small range of all ethical issues that are conceivable. Regarding the general ethical debate about smart glasses, ethical issues are related to privacy, safety, justice, change in human agency, accountability, responsibility, social interaction, power and ideology (23). The specific context of intraoperative support, however, is usually not considered in such generic analyses (24). Therefore, as sufficient preliminary work has been done, now is the right time to assemble the pieces into a comprehensive picture of ethical issues.

The aim of this systematic review is to determine systematically the full spectrum of ethical issues that is raised for surgeons in the intraoperative application of MR/AR technology. To this end, we performed a systematic review of ethical issues. Our research question is: Which *ethical issues* derive from the intraoperative application of MR/AR technology for surgeons?

## 2 Methods

### 2.1 Search strategy

**Figure 1:**
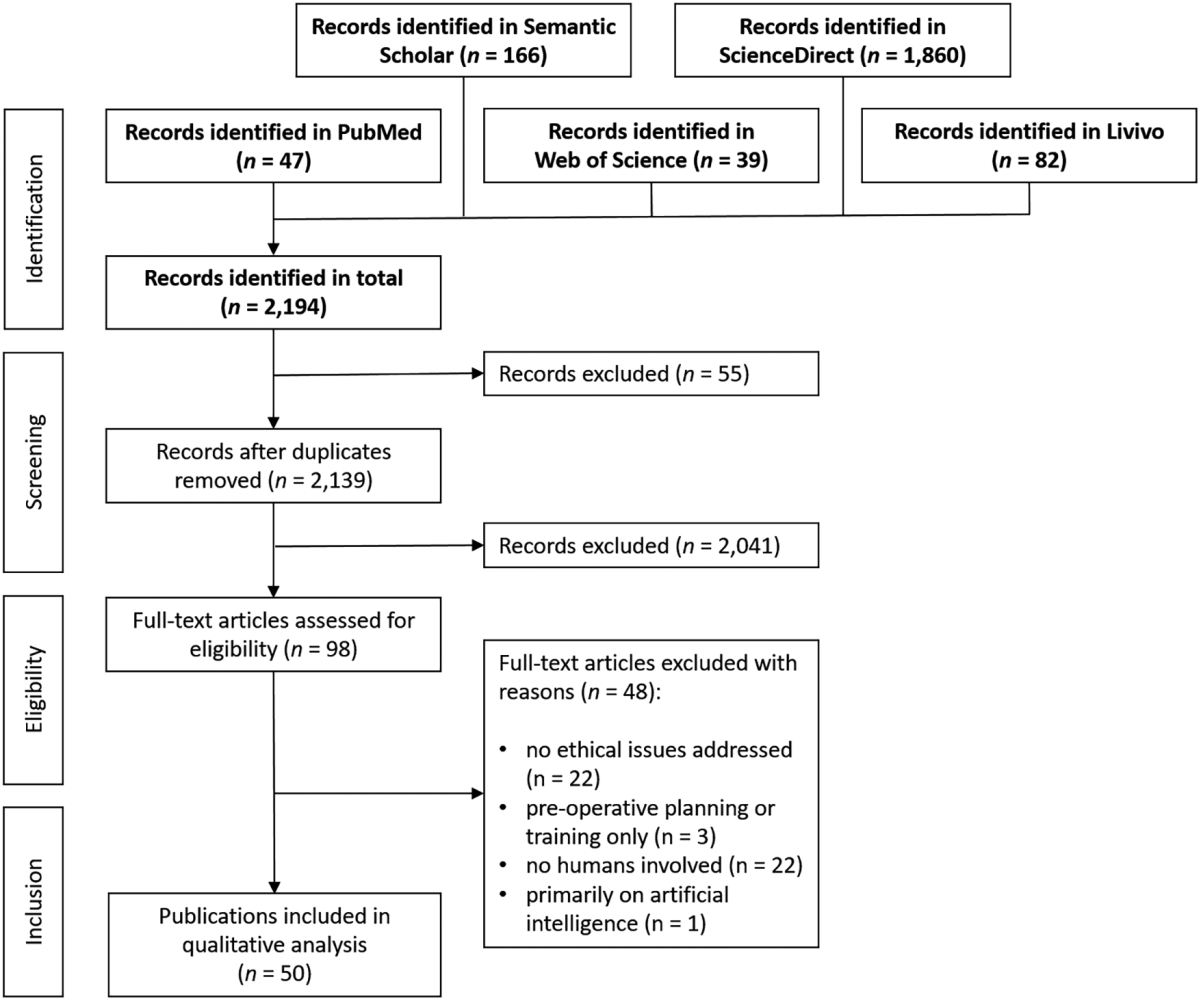
Flowchart of search strategy.

We report our systematic review according to the RESERVE statement (REporting of SystEmatic ReViews in Ethics, formerly PRISMA-Ethics) (28) (see Figure 1 and Table 1, and Supplementary S4). The search in five bibliographic databases on February 7, 2023, yielded *n* = 2,194 results, specifically in the Web of Science core collection *n* = 39, PubMed *n* = 47, Livivo *n* = 82, Semantic Scholar *n* = 166 and Science Direct *n* = 1,860 records. No restrictions on language, time span or article type were made for the searches in Web of Science, PubMed and Livivo. The search in Semantic Scholar was restricted to the subject area “medicine.” Due to the vast amount of results without restrictions in Science Direct, this search was restricted to the subject area “medicine and dentistry,” the time span of 2016–2023, as well as the article types of research articles, review articles, book chapters, case reports and practice guidelines. Due to export restrictions in ScienceDirect, bibliographic data was exported in bundles of 100 results.

**Table 1:** Search strings for bibliographic database search on February 7, 2023.

Total (*n* = 2,194)	
PubMed (*n* = 47)	(“hololens”[All Fields] OR (“smart glasses”[MeSH Terms] OR (“smart”[All Fields] AND “glasses”[All Fields]) OR “smart glasses”[All Fields] OR (“google”[All Fields] AND “glass”[All Fields]) OR “google glass”[All Fields]) OR (“hologram”[All Fields] OR “holograms”[All Fields] OR “holograms”[All Fields]) OR (“holographic”[All Fields] OR “holographical”[All Fields] OR “holographically”[All Fields]) OR ((“augment”[All Fields] OR “augmentation”[All Fields] OR “augmentations”[All Fields] OR “augmented”[All Fields] OR “augmenting”[All Fields] OR “augments”[All Fields] OR (“mixed”[All Fields] OR “mixes”[All Fields] OR “mixing”[All Fields] OR “mixings”[All Fields])) AND (“realities”[All Fields] OR “reality”[All Fields])) OR “augmented reality”[MeSH Terms]) AND “surg*”[All Fields] AND “ethic*”[All Fields]
Web of Science (*n* = 39)	ALL = ((((hololens) OR (google glass) OR (hologram) OR (holographic) OR (((augmented) OR (mixed)) AND (reality))) AND (surg*) AND (ethic*)))
Livivo (*n* = 82)	((((hololens) OR (google glass) OR (hologram) OR (holographic) OR (((augmented) OR (mixed)) AND (reality))) AND (surg*) AND (ethic*)))
Semantic Scholar (*n* = 166)	[(augmented OR mixed) AND reality] AND surgery AND ethics
ScienceDirect (*n* = 1,860)	[(augmented OR mixed) AND reality] AND (surgery OR surgical) AND (ethics OR ethical)

### 2.2 Selection process

After all records had been imported to the reference management software Endnote 20.1 (Clarivate), duplicates were removed, leaving *n* = 2,139 records. While screening titles and abstracts by one author independently (FU), *n* = 2,041 articles that dealt with virtual reality, medical education, surgical training, simulation or planning, robot involvement, diagnosis, veterinary medicine or mere technological study designs were excluded. Regarding the language of the articles, those not in English, German, Spanish, Dutch, French or Italian could not be assessed due to the language constraints of the authors. The selection process resulted in *n* = 98 records.

### 2.3 Eligibility criteria

Inclusion criteria for assessing the eligibility of full-text articles retrieved (*n* = 98) were the intraoperative application of MR or AR technology, the use of an HMD or any other display technology, such as microscopes, exoscopes, endoscopes, head-up displays or projections, and that ethical issues are addressed in respective publications. We included clinical studies (prospective and retrospective), casuistries, reviews, commentaries and opinion pieces. Exclusion criteria concerned articles that did not refer to ethical issues of MR/AR technology (*n* = 21), which referred only to preoperative planning or training (*n* = 3), which conducted studies only on phantoms, cadavers, 3D printed models or simulators so that no sentient humans were involved (*n* = 22), and which dealt primarily with artificial intelligence (*n* = 1). After the application of the eligibility criteria, *n* = 50 articles have been included in the data extraction process (see Supplementary S2).

### 2.4 Data extraction

**Figure 2:**
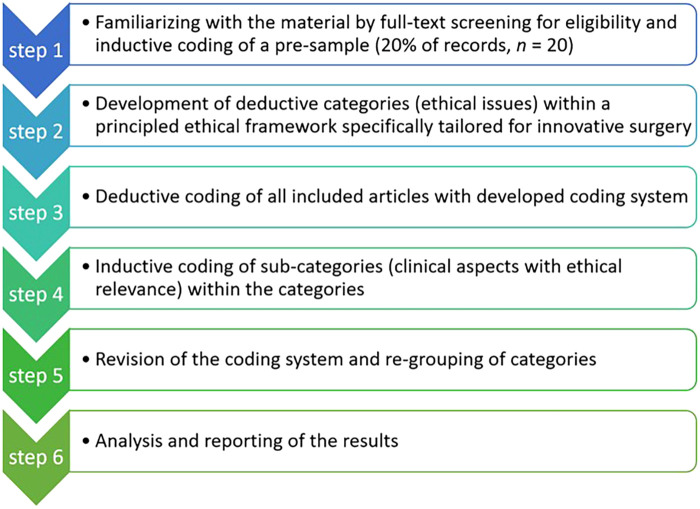
The methodological procedure of “content structuring content analysis” according to Kuckartz (29), adapted to this work.

We used the methodology of “content-structuring content analysis” according to Kuckartz (29) to extract ethical issues from the literature included (Figure 2). This method relies on a qualitative content analysis with an initial inductive coding of a pre-sample, the application of preselected deductive categories, and a final revision of the coding system to develop themes and sub-themes. We used the MAXQDA software for the coding process.

### 2.5 Identification of categories

In a first step, we conducted an initial mapping of ethical issues through inductive coding of the first 20% (*n* = 20) of all publications retrieved for full-text assessment (*n* = 98). One author (FT) with an academic background in medicine, social sciences and philosophy inductively coded this pre-sample independently to identify categories for ethical issues. This step aimed at generating use case-specific categories for ethical issues that were not influenced by the deductive categories of step 2 (see below). Two authors (CT, FU) reviewed and revised these categories by merging them to develop a preliminary coding system resulting in seven categories: (1) overreliance on new technologies and false safety, (2) proper application area for new technologies, (3) ensuring the technical expertise of surgeons, (4) facilitating access to healthcare, (5) responsibility to not withhold promising new treatment, (6) informed consent and data protection, and (7) the need for a national or international legal framework.

**Figure 3:**
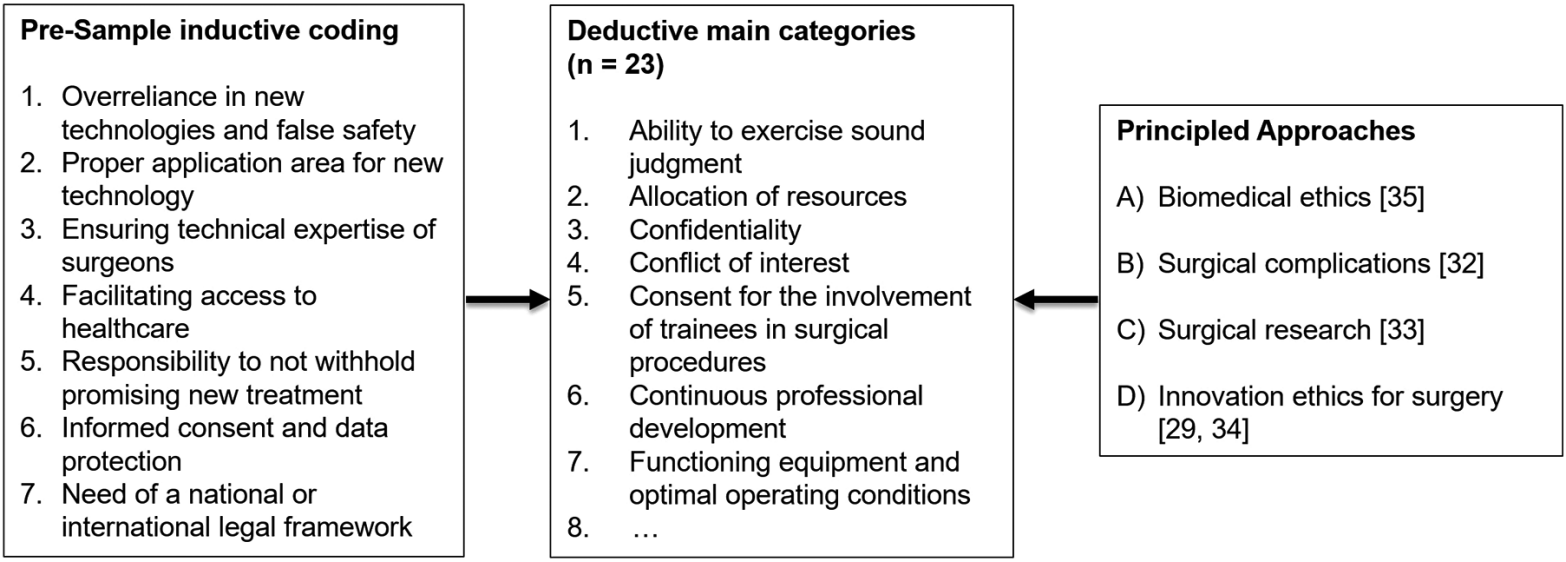
Development of deductive main categories for ethical issues in digital surgery.

In step two, we added deductive main categories to the inductively derived pre-sample categories with a synthesis of principle-based ethical frameworks for innovative surgery (see Figure 3). These ethical frameworks build on common ethical issues, which are the information units in which we are interested. Ethical issues in innovative surgery can usually be categorized by their source or motivation (e.g., economic incentives, epistemic shortcomings, regulatory failings) or by the agent or stakeholder that is affected (e.g., patients, surgeons, hospitals, healthcare or public health generally) (30). According to a common understanding, we define ethical issues as a violation of one principle or a conflict between at least two ethical principles, for example, respect for autonomy, beneficence, non-maleficence and justice (31). Accordingly, ethical issues emerge in situations in which people have difficulties to decide what should be done because there is a challenge in respecting one or more principles. Examples of ethical issues in surgical innovation include: not avoiding or minimizing foreseeable risks and harms; issues of data privacy in digital surgery; and potentially unintended long-term effects on human labor when using MR/AR technology versus improvements in safety and efficiency (32).

Ethical frameworks for surgical ethics rely on virtue ethics or casuistry approaches (31), principled approaches for surgical complications (33), surgical research (34) and innovation in surgery (30, 35). Because none of these approaches cover our use-case sufficiently (implementation of an innovation, not only research), we pooled them for our data acquisition and analysis. We selected *n* = 23 categories of ethical issues from the respective ethical frameworks as our deductive main categories within the structure of the widely acknowledged four principles of biomedical ethics (see Supplementary S1): respect for autonomy, beneficence, non-maleficence and justice (36). We additively integrated crucial aspects of innovation ethics for surgery to the ethical issues of surgery and surgical complications (31, 33) in order to cover the specifics of implementing novelties in the clinical workflow (30, 35) (Figure 3). Specifically, the ethical issues in connection with surgical innovation are “risks to patient safety, issues of informed consent and shared decision-making, cost considerations, conflicts of interest, […] threats to professionalism” (35) and “unfair allocation of healthcare” (30).

In step 3, we deductively coded the full material with the *n* = 23 deductive main categories. A category has been assigned to a text passage not only if a respective ethical issue was explicitly mentioned (semantic coding), but also when it was referred to implicitly (latent coding) (37). This coding was performed by one author (FU) and reviewed by another independently (FT). The material did not contain ethical issues of *n* = 5 categories: disclosure and discussion of surgical complications including medical errors, respecting human rights, respecting patient's requests (for procedures/particular surgeons), shared decision-making, and whistle-blowing.

In step 4, one author (FT) revisited the deductively coded categories of ethical issues from step 3 and inductively coded subcategories. This step aimed at achieving a finer granulation at the level of clinical and technical aspects that are the (bottom-up) real-world basis for ethical issues (instead of top-down ethical principles). Regarding the clinical aspects, we identified which errors, problems and risks can occur while using MR/AR technology intraoperatively and which may have ethical significance.

In step 5, the code revision of step 4 was reviewed by two authors (FU, LB) independently. Both authors then revised the whole coding system and grouped the categories again into themes (*n* = 10) and sub-themes in order to streamline the coding system (see Table 2). All authors revised these themes and inconsistencies were resolved by discussing the questionable cases until a consensus was reached.

**Table 2:** Comprehensive code system of themes, sub-themes and ethical issues.

1 Patient-physician relationship	1.1 Confidentiality	1.1.1 Ensuring	1.1.1.1 … privacy
			1.1.1.2 … cybersecurity
			1.1.1.3 … patient data is not being sold to companies without consent
			1.1.1.4 … only entitled users are granted access to patient data
	1.2 Awareness of	1.2.1 … the patient's perspective towards MR/AR	
		1.2.2 … reimbursing patients for using their data	
	1.3 Ensuring	1.3.1 … truth-telling	
		1.3.2 … trust within patient-physician relationship	
2 Informed consent	2.1 Information	2.1.1 Ability to explain digital surgery to patients	
	2.2 Obtaining consent for	2.2.1 … risks of MR/AR technologies	
		2.2.2 … involving trainees in surgery	
		2.2.3 … processing of patient data	
		2.2.4 … telesurgery	
3 Professionalism	3.1 Continuous professional development	3.1.1 Opportunity	3.1.1.1 … of access to experts world-wide (telementoring)
			3.1.1.2 … to avoid negative training (inappropriate behaviors)
		3.1.2 Ensuring	3.1.2.1 … legal literacy
			3.1.2.2 … AI literacy
			3.1.2.3 … data literacy
			3.1.2.4 … technical literacy
			3.1.2.5 … improvement of the learning curve
	3.2 Surgical competence	3.2.1 No standard of certifying digital surgery training	
		3.2.2 Inexperienced surgeons use MR/AR	
		3.2.3 Ensuring thorough operation planning	
		3.2.4 Recognizing the limits of one's professional competence	
		3.2.5 MR/AR enables new maneuvers for surgeons	
4 Research and innovation	4.1 Need for	4.1.1 … continuous research and auditing	
		4.1.2 … comparative studies evaluating clinical endpoints	
		4.1.3 … active participation in developing MR/AR to be successful	
		4.1.4 … evaluation studies through user assessment	
		4.1.5 … definition of the new gold standard (2D vs. 3D)	
		4.1.6 … cost-effectiveness studies	
	4.2 Innovation anxiety	4.2.1 Mitigating resistance against innovation	
		4.2.2 Recognizing the fact that MR/AR is a technology under development	
	4.3 Research ethics	4.3.1 Demand for more and larger RCTs before implementation	
		4.3.2 Opt-out option for patients regarding use of their data	
		4.3.3 Evidence must demonstrate clear advantages	
		4.3.4 Debated ethicality of control groups in surgical research	
5 Legal and regulatory issues	5.1 Data protection	5.1.1 Issues of data protection and data ownership	
	5.2 Liability	5.2.1 Unclear liability of surgeons who do not follow decision support	
		5.2.2 Surveilled surgeons fear litigation in medical negligence cases	
	5.3 Standard Procedures	5.3.1 Need for regulatory framework for clinical trainees	
		5.3.2 Lack of standardization	
		5.3.3 No standard operating procedures for proper patient consent	
		5.3.4 No standard for informed consent	
6 Functioning equipment and optimal operating conditions	6.1 Ensuring	6.1.1 … improvement of comfort, ergonomics, and usability of device	
		6.1.2 … awareness of the limitations of MR/AR technology	
		6.1.3 … hygienic requirements like sterile equipment	
		6.1.4 … surgeon's motion, peripheral vision, and general perception	
		6.1.5 … the ability to toggle the HUD on/off to avoid distraction	
		6.1.6 … accuracy of super-imposed images	
		6.1.7 … evidence-based implementation	
	6.2 Mitigating the risk of	6.2.1 … registration errors	6.2.1.1 … due to auto-registration
			6.2.1.2 … due to re-registration
			6.2.1.3 … due to deformation of soft tissues
			6.2.1.4 … due to position shifts
			6.2.1.5 … due to inadvertently touching the reference frame
		6.2.2 … segmentation errors	
		6.2.3 … tracking errors	
		6.2.4 … obscuration of the operating field	
		6.2.5 … impaired accuracy	
		6.2.6 … delayed reaction time of the equipment (latency)	
		6.2.7 … decreased usability	
		6.2.8 … attention shift and dissociation	
		6.2.9 … inattentional blindness	
		6.2.10 … decreased acceptability due to laborious adjustments	
	6.3 Need for maintaining redundant standard procedures (dead loss)	6.3.1 … due to patient safety protocols	
		6.3.2 … due to loss of internet connection	
		6.3.3 … due to dead loss ("blue screen")	
		6.3.4 … due to memory issues	
		6.3.5 … due to battery issues	
	6.4 Mitigating health risks for users of MR/AR technology like	6.4.1 … cybersickness, motion sickness, vertigo, and nausea	
		6.4.2 … headache	
		6.4.3 … ophthalmic syndromes	
		6.4.4 … discomfort	
7 Allocation of resources	7.1 Reduced costs	7.1.1 … due to higher number of procedures executed in the same time	
		7.1.2 … through in-house development compared to imported devices	
	7.2 Increased costs	7.2.1 … while little increase in benefits	
		7.2.2 … due to expensive equipment	
		7.2.3 … due to high set-up costs	
		7.2.4 … due to presence of additional personnel	
		7.2.5 … due to 3D model work	
		7.2.6 … due to expensive training personnel	
8 Minimizing harm	8.1 Reducing	8.1.1 … invasiveness	
		8.1.2 … radiation exposure	
		8.1.3 … procedure time	
		8.1.4 … task load and cognitive load of surgeons	
	8.2 Awareness	8.2.1 … when critical structures are threatened	
		8.2.2 … of high-risk clinical intervention	
		8.2.3 … of possible errors	
	8.3 Ensuring	8.3.1 … redundant conventional techniques as safeguardings	
		8.3.2 … better outcomes through research	
9 Good communication skills of surgeons	9.1 … with patients		
	9.2… with colleagues		
	9.3 … with technicians		
	9.4 … with companies		
10 Ability to exercise sound judgment	10.1 Decision-making	10.1.1 More certainty in decision-making	
		10.1.2 Avoidance of overreliance in new technology and false safety	
	10.2 Awareness of possible	10.2.1 … bias towards MR/AR hyped by marketing departments	
		10.2.2 … ethical issues	
		10.2.3 … complications like position shifts	
	10.3 Selection of the appropriate	10.3.1 … application area	
		10.3.2 … device for the intended purpose	
		10.3.3 … technology (AR, MR or VR)	
	10.4 Not withholding MR/AR if it performs better		

### 2.6 Synthesis methodology

Finally, we analyzed the code system in different dimensions and narratively synthesized the results at a high level of aggregation. Code frequencies (“How many codes appear in a document?”) and mention frequencies (“How often is a code mentioned in all documents?”) were documented. We also used MAXQDA's code matrix browser to determine the papers that contain the most code mentions. We also provide the code system with anchor examples in Supplementary S3 and a narrative summary of the most important findings in the results section below. Being aware that the number of mentions of an ethical issue does not necessarily correspond to its clinical importance, we only report those ethical issues that meet at least one of the following criteria: uniqueness, relevance or importance (according to the experience and judgement of the authors) as well as representing a crosscutting theme over several main categories. For the latter, we used MAXQDA's code relation browser to identify segment overlaps and, thereby, co-occurring ethical issues.

## 3 Results

### 3.1 Sample description

The *n* = 50 publications included in this systematic review were published in a heterogeneous pool of medical journals predominantly related to surgery (*n* = 28), orthopedics (*n* = 5) and urology (*n* = 4). The journals with the most publications included are “World Neurosurgery” (*n* = 4), the “Annals of Surgery” (*n* = 3), the “Journal of Oral and Maxillofacial Surgery” (*n* = 3) and “European Urology” (*n* = 3). The articles were published between 2012 and 2023, the majority in 2021 (*n* = 16) and 2019 (*n* = 11). While the publications originated from worldwide sources, mostly from the US (*n* = 9), Germany (*n* = 7) and Italy (*n* = 5), most of the texts were written in English, with only a few of them being partly or completely in German (*n* = 2) or Spanish (*n* = 1). Many of the publications included were reviews (*n* = 12) and original research (*n* = 5), although the type of article was often unspecified.

### 3.2 Ethical issues and code frequencies

We found *n* = 143 ethical issues pooled in ten main themes with *n* = 666 mentions in total. The five most prevalent ethical issues are “Need for continuous research and innovation” (*n *= 24), “Ensuring improvement of the learning curve” (*n* = 21), “MR/AR enables new maneuvers for surgeons” (*n* = 18), “Ensuring improvement of comfort, ergonomics, and usability of devices” (*n* = 18) and “Not withholding MR/AR if it performs better” (*n* = 18). These five issues have also been addressed most often within individual publications. The three most prevalent main themes are “Functioning equipment and optimal operating conditions” (*n* = 191), “Ability to exercise sound judgment” (*n* = 99) and “Professionalism” (*n* = 96). The three least prevalent themes are “Informed consent” (*n* = 23), “Good communication skills” (*n* = 25) and “Legal and regulatory issues” (*n* = 33). The three articles with the most mentions of ethical issues are Lam et al. (*n* = 42) (4), Benmahdjoub et al. (*n *= 37) (38) and Sakai et al. (*n *= 35) (14). Crosscutting ethical issues that are strongly connected to other ethical issues are research and auditing, comfort and usability, awareness of the technological limitations, accessibility, privacy, learning curve, accuracy, error mitigation and economic issues (Table 3).

**Table 3:** Crosscutting ethical issues that have a strong connection in terms of segment overlaps according to MAXQDA's code relation browser.

Ethical issue in connection with …	… other ethical issues
“Ensuring privacy”	“Ensuring cybersecurity” “Obtaining consent for processing of patient data” “Awareness of possible errors” to minimize harm
“Ensuring continuous research and auditing”	“Need for comparative studies evaluating clinical endpoints” “Ensuring better outcomes through research” to minimize harm several aspects of ensuring “Functioning equipment and optimal operating conditions” “Good communication” with colleagues, technicians and companies “Selection of the appropriate application area”
“Ensuring improvement of comfort, ergonomics and usability of devices”	“Mitigating the risk of attention shift and dissociation” “Ensuring awareness of the limitations of MR/AR technology” “Mitigating health risks for users of MR/AR technology like cybersickness, motion sickness, vertigo, and nausea” “discomfort”
“Ensuring awareness of the limitations of MR/AR technology”	“Mitigating the risk of impaired accuracy”

### 3.3 Themes and sub-themes of ethical issues

#### 3.3.1 Patient-physician relationship

The ethical issues related to the patient-physician relationship can be categorized into the following sub-themes: confidentiality, awareness of the patient's perspective and reimbursing patients for using their data, as well as ensuring truth-telling and trust. To safeguard confidentiality, it is imperative to implement measures that protect privacy and enhance cybersecurity, thereby, thwarting unauthorized access to patient data. Additionally, obtaining patient consent for any utilization of their data is crucial to ensure ethical compliance.

**Examples**: Six papers contain a high number of mentions (*n* ≥ 3) (4, 13, 39–41), while concerns about privacy (*n* = 12) and cybersecurity (*n* = 11) are most frequently mentioned. These issues belong to confidentiality and are linked to legal and regulatory requirements that mandate the protection of patient data and the prevention of unauthorized access to it:

Ethical aspects are also a major consideration when investigating new devices for surgical practice. Such technology not only raises concerns for data privacy and protection but should also guarantee accuracy, safety and security for a potential use in patients. According to the European Union's General Data Protection Regulation (GDPR), the patient's consent is a main concern for the use HMD or smart glasses, as the device processes medical information. Besides, the device needs to be configured to protect the patients' data (13).

A little noticed but important issue is the concern that patients are not reimbursed for using their data (*n* = 2):

Data sharing encompasses sharing of data including between different technologies, between hospitals, and between hospitals and commercial partners. […] there are currently no guidelines concerning data ownership, and the international legal requirements concerning data sharing are unclear (4).

#### 3.3.2 Informed consent

The ethical issues surrounding informed consent are concerned primarily with the ability to provide patients with accurate and understandable information about digital surgery and obtain their consent for its use. Specifically, the risks associated with MR/AR technologies, such as the involvement of trainees in surgery, the processing of patient data and the use of telesurgery, must be clearly explained to patients in order to obtain informed consent.

**Example**: One paper contains by far the most mentions (*n* = 7) (3) and provides specific information to be disclosed to patients concerning the processing of their data (*n* = 12):

Although digital surgeons will probably remain accountable for the decisions that they make, it is now possible that they will also have to contend with automation bias, opaque algorithms, and a rapidly evolving ecosystem of sophisticated cloud-based platforms and connected hardware. This will in turn create new challenges for consent and litigation, which are as of yet untested (3).

#### 3.3.3 Professionalism

One major ethical issue is the need for continuous professional development, which can be facilitated by access to global experts through telementoring and the avoidance of negative training outcomes. Ensuring legal, artificial intelligence, data, and technical literacy is crucial for healthcare professionals to remain competent in the use of MR/AR technology and improve their learning curves. Another issue is surgical competence, where inexperienced surgeons may use MR/AR without standardized certification of digital surgery training. To address this, it is necessary to ensure thorough operation planning, recognize the limits of one's own professional competence and enable new maneuvers for surgeons.

**Examples**: Three papers contain a high number of mentions (*n* ≥ 7) (4, 42, 43), while new maneuvers for surgeons (*n* = 34), ensuring to improve the learning curve (*n* = 27), and ensuring artificial intelligence literacy (*n* = 10) are most frequently mentioned.

“Binary surgeons” who do not have access to digital robotic platforms or who choose to reject them will be left in a perilous position where their performance will be compared with digitally augmented colleagues regardless of whether they “opt out.” (3)

There is a steep learning curve in integrating this technology for new adopters, especially for those with limited immersion in AR, VR or MR environments. This carries a potentially high cost in training personnel with few technologies on the market holding an oligopoly (14).

Developing a solution that provides high accuracy in terms of registration (technology metrics) does not necessarily make it more efficient when utilized during surgery (surgery outcome). For instance, a single user might perform poorly in the case that they are not familiar with the technology employed (38).

#### 3.3.4 Research and innovation

Continuous research and auditing, comparative studies and user assessments are necessary to evaluate clinical endpoints, define new standards and assess cost-effectiveness in order to ensure the safe and effective use of MR/AR technology. Healthcare providers must actively participate in developing MR/AR technology to ensure success. Innovation anxiety needs to be addressed, including the recognition that MR/AR is a technology under development and the need to mitigate resistance against innovation. Ethical concerns related to research must also be addressed, including the need for more and larger randomized controlled trials, an opt-out option for patients regarding the use of their data, clear demonstration of advantages and debates over the ethicality of control groups in surgical research.

**Examples**: Two papers contain a high number of mentions (*n* ≥ 8) (38, 44). In addition to the major issue of the need for continuous research and innovation (*n* = 40), the ethicality of control groups has been debated:

The limitation of our study was its retrospective nature and that no control group, such as a series of patients with previous surgery or anatomical variants undergoing transsphenoidal surgery without AR support, was included. However, considering the reported numbers of severe complications during transsphenoidal surgery of <1%–2% depending on the surgeon's experience, a comparative study to prove the patient safety benefit of AR would be unethical or unpractical. In such a study, surgery would either have to be performed by an inexperienced surgeon to encounter severe complications, or, in the case of an experienced surgeon, the case numbers required to prove additional safety might be too large (42).

#### 3.3.5 Legal and regulatory issues

One major issue is data protection and ownership, which must be addressed to ensure patient confidentiality. Liability is another major concern, as the unclear liability of surgeons who do not follow decision support and the fear of litigation among surveilled surgeons in medical negligence cases pose significant ethical challenges. Standardization is also lacking, with a need for a regulatory framework for clinical trainees, standard operating procedures for proper patient consent and a standard for informed consent.

**Examples**: Two papers contain a high number of mentions (*n* ≥ 7) (3, 4). Issues of data protection and data ownership were the most frequent (*n* = 10):

We did not identify any data investigating patients' views on XR-assisted surgery. Instead, there has been a greater focus on the cost, reliability, and feasibility of these technologies to establish their place in surgery, when compared to studies published in the early 2000s. The patient viewpoint is of particular relevance due to increased international scrutiny on how patient's data is used and how their privacy is protected and requires further research (20).

#### 3.3.6 Functioning equipment and optimal operating conditions

Functioning equipment and optimal operating conditions are ethically relevant due to the tensions between following the ethical principles of beneficence and non-maleficence, i.e., by seeking to continuously improve treatment while reducing various possible risk factors. This theme contains the largest quantity of ethical issues, including the need for ensuring improvements in comfort, ergonomics and the usability of the device, awareness of the limitations of MR/AR technology, and evidence-based implementation. Hygienic requirements, the accuracy of superimposed images, ensuring surgeon's motion, peripheral vision and general perception, as well as the ability to toggle the HMD on and off to avoid distraction, are also important factors.

However, there are risks associated with registration errors, segmentation errors, tracking errors, obscuration of the operating field, impaired accuracy, delayed reaction time of the equipment (latency), decreased usability, attention shift and dissociation, inattentional blindness, decreased acceptability due to laborious adjustments, and health risks for users of MR/AR technology. It is necessary to maintain redundant standard procedures through protocols to ensure patient safety and mitigate risks, to address the loss of internet connection, dead loss (“blue screen”), memory and battery issues. Additionally, mitigating health risks for users of MR/AR technology, such as cybersickness, motion sickness, vertigo, nausea, headache, ophthalmic syndromes and discomfort is crucial for successful operations.

**Examples**: Five papers contain a high number of mentions (*n* ≥ 10) (14, 45–48).

To implement automatic registration, deformation factors must be considered, including intraoperative movement caused by the surgeon and the instruments, as these can also produce displacement. […] otherwise surgical progress can be delayed considerably, increasing the risk of intraoperative complications (49).

Another concern is related to the ergonomics and the comfort of the users. HMD devices are still heavy […]. This can cause discomfort and fatigue to the user, especially for long surgical procedures. […] Several users also report cybersickness such as nausea, visual discomfort, dizziness, headaches, eye strains or dry eyes. An adjustment period and an appropriate training is also required for the operators to get familiar and use the device efficiently (13).

#### 3.3.7 Allocation of resources

The principle of justice is represented by issues of resource allocation, including increased costs due to expensive equipment and training. The implementation of MR/AR technology could potentially lead to reduced costs through a higher number of procedures executed in the same time, as well as through in-house development compared to imported devices. However, costs may also increase due to expensive equipment, high setup costs, the presence of additional personnel, 3D model work, and expensive training of personnel, while providing little increase in benefits.

The MR/AR-based devices enable healthcare professionals to perform surgical procedures across spatial distances and, thereby, allow patients to access the best surgical care and expertise worldwide via telementoring. This touches on the principle of social justice and questions related to ethical challenges with global health. Telesurgery interventions can support the transfer of surgical expertise from highly specialized healthcare facilities to remote areas with less expertise, leading to the better availability of optimized healthcare services worldwide.

**Examples**: One article had by far the most mentions (*n* = 10) (14). The most frequent concern was increased costs while little increase in benefits (*n* = 13):

Finally, the economic aspect is another concern. The cost of wearable technologies is variable depending on the device, the manufacturer and the software implemented. The cost of commercialized devices currently available ranges from 1,000 to 3,000 euros for smart glasses and from 350 euros to 3,500 euros for HMD. It mainly depends on the need of the user and its intended applications. The cost for implementation and maintenance of such systems would have to be put in balance with the clinical benefits before hoping to expand its use in common surgical practice (13).

Five papers addressed the topic of improved access to surgical expertise and improved health services. Two of them specifically highlighted potential avenues for a more equitable distribution of healthcare around the world, while three papers generally noted improved access to surgical expertise via telemonitoring:

As well as guiding surgeons in theatre, AR systems have the potential to enhance preoperative planning and training. The new worlds of “telepresence” and “telementoring”, both supported by AR technologies, may become invaluable tools for teaching and training across wide geographical boundaries and may increase access to expert clinical opinion for patients world-wide (50).

#### 3.3.8 Minimizing harm

The ethical issues related to minimizing harm can be summarized in three sub-themes. Firstly, there is a need to reduce the invasiveness, radiation exposure, procedure time, task load and cognitive load of surgeons. Secondly, awareness is crucial to recognize high-risk clinical interventions, possible errors and when critical anatomical structures are threatened. Thirdly, ensuring better outcomes through research and having redundant conventional techniques as safeguarding measures is essential.

**Examples**: Two papers had by far the most mentions (*n* ≥ 7) (51, 52). The most frequent concerns were ensuring better outcomes through research (*n* = 21) and reducing the radiation exposure (*n* = 11) because some registration techniques need scans:

For procedures in the mid-thoracic region, relatively long scan ranges are required to ensure reliable nonlinear registration with the preoperative images. Further dose reduction might be possible, until a threshold is reached in which the lowered resolution and increased image noise prevent reliable nonlinear image registration. Radiation-free alternatives for registration are available; however, they do not provide the same accuracy as iCT-based registration. Surface matching techniques such as using a navigation pointer can only be used reliably for a single level and are not implemented for anterior, lateral, or paravertebral approaches (53).

#### 3.3.9 Good communication skills

Surgeons need to interact appropriately with patients, colleagues, technicians and companies, therefore, they need good communication skills and channels.

**Examples**: Two papers contain the most mentions (*n* ≥ 4) (4, 50) while good communication with commercial companies was the most frequent issue (*n* = 9):

Panellists agreed that there is a lack of framework or experience within the majority of institutions for the setting up of fair partnerships between healthcare and commercial entities. They highlighted issues surrounding inequality of power and differing motives between hospitals and commercial companies. Finally, panellists agreed that commercial partnerships may result in restriction on the ability of hospitals to report results (4).

#### 3.3.10 Ability to exercise sound judgment

The ability to exercise sound judgment is a critical ethical issue because surgeons must be able to assess all disease-relevant conditions of the human body and select the appropriate medical interventions on this basis. The selection of the appropriate application area, device and technology (AR, MR, VR) is essential for avoiding overreliance on new technology and false safety. The awareness of possible bias towards MR/AR hyped by marketing departments, ethical issues and complications, such as position shifts, is crucial. The use of MR/AR technology must not be withheld if it performs better, and more certainty in decision-making can be achieved.

**Examples:** One paper contains by far the most mentions (*n* = 10) (54), and the most common concern was that MR/AR should not be denied if it provides better outcomes than standard procedures (*n* = 29). The second frequent concern was the avoidance of overreliance on new technology and false safety:

There is often a mindset driven by curiosity and wonder that any new tool will, in a manner of speaking, ‘change the game.’ For plastic surgeons, there must always be thoughtfulness behind using technology, backed by evidence to support its use (55).

## 4 Discussion

**Figure 4:**
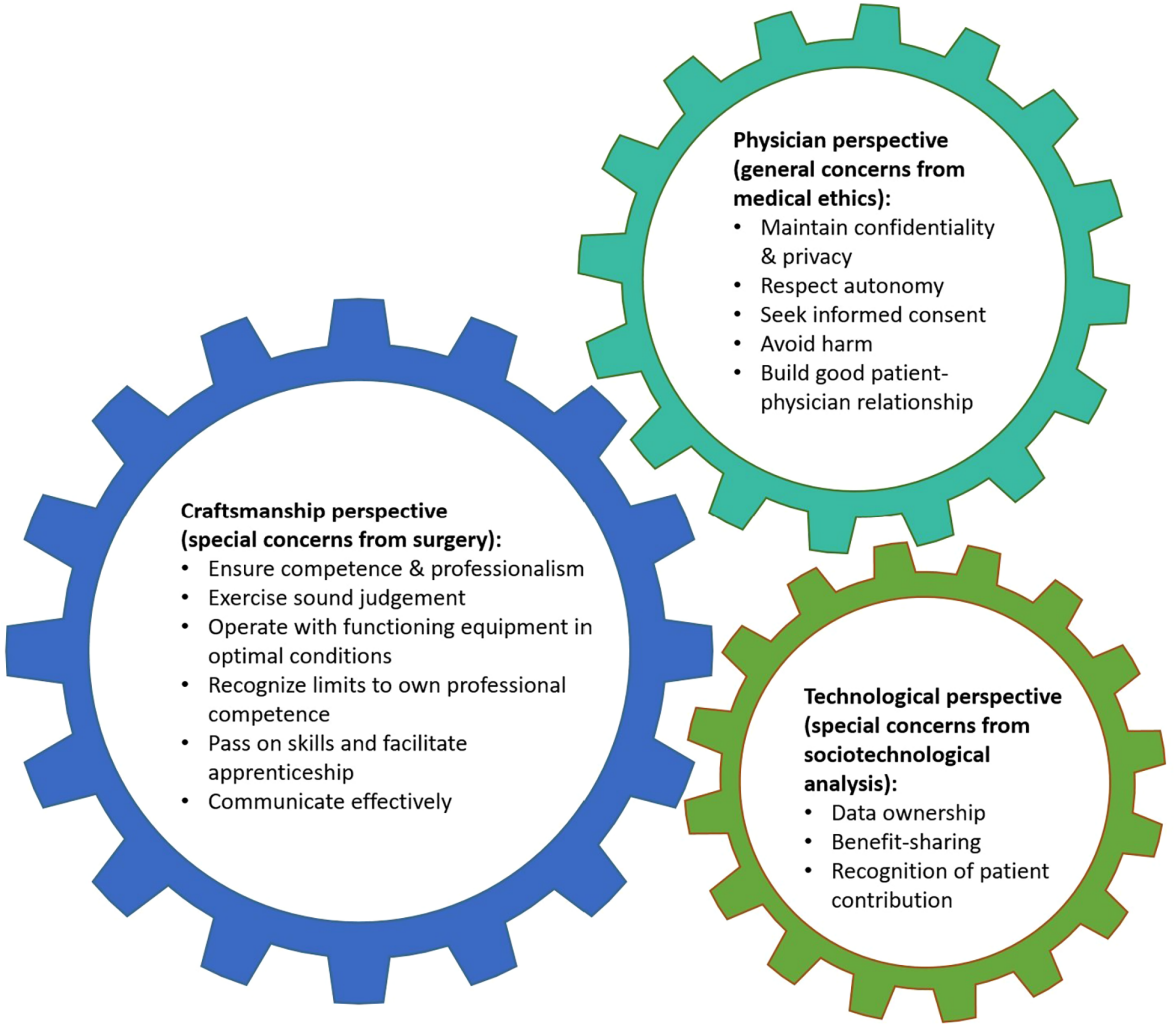
The surgeon's new ethical toolbox.

The literature we have analyzed has a clear emphasis on clinical aspects, particularly the errors, problems and risks that can occur while using MR/AR technology intraoperatively. Yet, despite dealing with forefront innovation, we can distinguish two sets of ethical issues that can be best understood in relation to the two historical roots of surgery: the surgeon as a craftsman and as a physician (56). A third set of ethical issues examined in the literature are specific to technologies that use a large amount of sensitive data. We will discuss these three sets of ethical issues in turn, while we also know that these are sociotechnical applications of digital health (57) (see Figure 4).

### 4.1 Surgeon as a craftsman

A central aspect of the literature dealt with how a surgeon could or even should embody the virtues of good craftsmanship: to possess a high level of competence, expertise, judgment and good communication with peers. This includes upholding professionalism, ensuring competence, exercising sound judgment, passing on skills and facilitating apprenticeship, effectively communicating and working in optimal conditions with functioning equipment (58).

Achieving good results is central in a professional tradition that fully acknowledges the importance of developing skills and putting such skills at the service of others. Based on this background, a recurrent worry when balancing the advantages of new technologies with the risks of becoming dependent upon them is epitomized in the question “What if it doesn't work?” Surgeons as craftsmen are well aware that they will need to adapt their plans to unforeseen events and are keen to maintain their liberty and capacity to act according to their best professional judgement swiftly. This is rarely clearer than with surgery, where adapting procedures quickly and efficiently is an essential skill to avoid further harm or even death.

The introduction of MR/AR technology introduces new challenges that may affect these aspects. Malfunctions of the technology, such as registration errors, tracking errors or obscured operating fields, can impact a surgeon's ability to perform optimally. This raises questions about the potential consequences of relying heavily on such technology and the need to maintain surgical competence and proficiency without overreliance on external aids. There are, however, at least four factors that can reduce risks.

Firstly, surgeons must undergo proper training and education to integrate these technologies effectively and safely into their practice. Ethical obligations exist to ensure that surgeons have the necessary skills and competencies to utilize MR/AR technology appropriately and responsibly, avoiding potential harm to patients. Surgeons must be prepared to act immediately when unforeseen circumstances arise during a procedure, such as unintended tissue damage or equipment failure. The urgency and time-sensitive nature of surgical interventions require surgeons to make quick decisions and adapt to evolving situations while considering the best interests and well-being of the patient. This challenge is addressed by our finding that surgeons should have the ability to exercise sound judgment in terms of recognizing the limitations of the intraoperative MR/AR application.

Secondly, a certain level of redundancy is needed. Having backup systems or alternative approaches in place to mitigate the risks of equipment failure or human error should become standard. This can include duplicate or backup devices, extra staff members with overlapping skills or contingency plans to address unforeseen circumstances. However, redundancy can also lead to additional costs, resource allocation challenges, and conflict with ethical principles of fairness and equity. It raises questions about the allocation of limited resources and whether the redundancy measures are justified in terms of benefits and risks.

Thirdly, acceptance of the technology among surgeons needs to be improved. This requires addressing the surgeons' fears of being continuously under surveillance. Ergonomic issues are also central, as surgeons are required to wear gear for long shifts, which may have implications for their own well-being and self-care. Ethical responsibilities extend to promoting the health and safety of surgeons and addressing the ergonomic challenges they face to prevent burnout and maintain their ability to provide quality care. This might be the explanation why there was so much consideration about optimal operating conditions in our material.

Lastly, the introduction of this new technology should ideally allow for the continuity of deeply anchored values within the profession that have their roots in good craftmanship. Surgery has a long-standing tradition of professional commitment to training new generations. Surgeons are responsible for passing down their knowledge and hands-on expertise to trainees, ensuring the continuous professional development and a steady learning curve. This tradition-based argument places ethical obligations on surgeons to fulfill their educational role and contribute to the professional development of future surgeons. Younger surgeons may be more inclined to utilize digital tools but, at the same time, are less experienced. This poses the risk of a digital divide in surgery, although there is an ethical imperative for surgeons to embrace new technological opportunities and work collaboratively to improve their craft.

### 4.2 Surgeon as a physician

Large parts of the literature addressed surgeons as specialist physicians who had undergone medical ethics training as part of their studies and further career development. These ethical issues were standard medical ethics concerns applicable to all medical specialties, such as maintaining confidentiality, respect for the patient's autonomy, informed consent and avoiding harm.

As the introduction of MR/AR technology is still in an experimental stage, there was a wide awareness of the need to inform patients about the nature of the new technology and the current uncertainties. Digital surgery stands apart from other medical specialties because new surgical techniques often cannot be tested through randomized controlled trials, as it may not be ethically feasible or practical to randomize patients (or experienced and inexperienced surgeons) for certain interventions. This poses a challenge if not constraints, generating strong empirical evidence for the efficacy and safety of novel surgical approaches (59).

Although the patient-physician relationship has rarely been addressed in our material, the introduction of MR/AR technology may alter the dynamic between patients and surgeons. Patients may have concerns regarding the technology and how it affects the surgeon's decision-making during operations. Surgeons must, therefore, communicate effectively—ensuring that they understand the implications, benefits and potential risks associated with the use of MR/AR technology—to obtain informed consent from patients. This may also require an exploration of the patients' perspective, expectations and preferences regarding surgical innovations such as MR/AR to foster transparency and trust. Patients who feel heard and involved in their care are more likely to trust their healthcare providers and feel confident in the treatment options recommended.

Similarly, ethical principles, such as beneficence and avoiding harm, were referred to explicitly and implicitly. There are several epistemic and practical limitations pertaining to the application of a technology in a high-risk context for a purpose for which it was not developed, and there is the risk of viewing the operating room as a “playground” for the trial and error of surgical innovation. A balance between improving general surgical outcomes by innovating and ensuring individual patient safety needs to be drawn. It emphasizes the need for thorough evaluation, evidence-based practice, continuous monitoring of outcomes and adequate communication skills for informed consent. Surgeons should have a comprehensive understanding of the limitations of the technologies they employ and adhere to ethical principles of respect for autonomy, non-harm and beneficence to ensure that patient welfare remains paramount.

### 4.3 Technology-specific ethical issues

The technology uses and harvests large amounts of data, thus, a few studies raised the question of the ethics of data ownership (60). Should there be some type of benefit-sharing among those patients who have contributed their data? Ethical considerations emphasize the importance of ensuring that patients who provide their data for research or the advancement of digital surgery technology are appropriately acknowledged and have a share in the benefits that result from these contributions. Furthermore, the commercialization of patients' data may raise issues of privacy. However, it remains unclear whether the demand for benefit sharing is more pronounced within surgery compared to other medical specialties. While the patient was often regarded as the owner of the data, this question is far from settled in medical ethics (60–62). Furthermore, technology-specific issues should be considered in the larger context as sociotechnical problems of digital health related to physical devices, interpersonal relationships, organizational policies, corporate contracts, and government regulations that shape how digital health technologies are adopted and used (57).

The sociotechnical challenges posed by digital health technologies manifest in a control dilemma, wherein a novel technology can only be properly regulated and its ethical implications anticipated after it has been implemented. Since several HMDs are already commercially available in connection with advanced software for intraoperative surgical applications there is urgency in anticipating the ethical implications at an early stage of their implementation.

## 5 Conclusions

This systematic review highlights the ethical complexities surrounding the intraoperative application of MR/AR technology in digital surgery for the near future. As the technology is still under development and its use is still limited, further ethical issues could arise if it is used more frequently. Some other ethical issues could then also be classified as non-critical. However, consideration of these ethical issues is relevant not only for HMDs due to the introduction of MR/AR technology, but especially for robotic surgical systems using minimally invasive approaches in the near future. The implementation of MR/AR technology in the operating room invites for reflections on the position of technology between the patient and the surgeon. Therefore, themes of ethical issues include the patient-physician relationship, informed consent, and confidentiality. Further themes of ethical issues such as professional competence, research and innovation, functioning equipment, resource allocation, minimizing harm, and sound judgment are aimed rather at the actions of surgeons. The exploration of these ethical issues underscores the need for comprehensive frameworks and guidelines to address the challenges posed by MR/AR technology in digital surgery. Ethical considerations surrounding confidentiality and data protection emphasize the importance of privacy safeguards, consent mechanisms and secure data management practices. An awareness of the patient's perspective, benefit sharing and the demand for evidence-based implementation contribute to responsible research and innovation. The literature also emphasizes the significance of maintaining professionalism, surgical competence and continuous professional development facing technological advancements. The challenges associated with ensuring functioning equipment, optimal operating conditions and minimizing errors necessitate ongoing vigilance and an adherence to standard procedures.

Moreover, the ethical dimensions of minimizing harm and exercising sound judgment highlight the importance of addressing ergonomic issues, potential risks and the selection of appropriate applications of new technologies. Balancing the advancement of surgical craft with the ethical safeguards, such as respect for autonomy, informed consent and patient-centered care, remains paramount. Overall, the focus on clinical aspects of MR/AR technology in surgery has shed light on the numerous ethical issues associated with surgery as a craft. It emphasizes the need to strike a balance between leveraging technological advancements and upholding the core principles of surgical practice, such as professionalism, competence, sound judgment and patient safety.

To give a positive outlook, the use of MR/AR-based technologies also holds potential advantages when it comes to a more equitable distribution of surgical expertise and optimized surgical healthcare services. Social justice, as a core principle of medical ethics, has been used to call for the availability of adequate healthcare services worldwide (63). Enabling surgeons to participate in telemonitoring or virtual coworking spaces with more experienced colleagues across regional or national borders may improve training conditions for future surgeons and, thereby, lead to a significant optimization of local surgical healthcare. In this context, a more democratized provision of healthcare might also be relevant in the perspective of global health justice. Transferring knowledge and surgical expertise from more developed regions in the world to remote areas may, therefore, also impact still enduring inequalities in global healthcare (64).

## Data Availability

Additional data of the study are included in the Supplementary Material, further inquiries can be directed to the corresponding author.
