# Supplementary material for: Intraoperative application of mixed and augmented reality for digital surgery: a systematic review of ethical issues
Source: Front Surg. 2024 Mar 14;11:1287218. doi: 10.3389/fsurg.2024.1287218 (PMC10972963; doi:10.3389/fsurg.2024.1287218)
Supplement: Supplementary file 1 [file Datasheet1.docx]

**Supplement 1.** Deductive categories for the first coding.

| **Main categories for the first deductive coding (ethical issues)^[[1]](#footnote-1)^** | **Definition** | **Overlapping categories resulting from the first inductive pre-sample coding** | **Relation to a principle of biomedical ethics^[[2]](#footnote-2)^** | **Has the category been assigned during the deductive coding?** |
| --- | --- | --- | --- | --- |
| 1. Ability to exercise sound judgment | Healthcare professionals must be able to assess all disease-relevant conditions of the human body and select the appropriate medical interventions on this basis. | facilitating access to healthcare via tele-surgery; overreliance on new technologies; ensuring technical expertise of surgeons | beneficence | yes |
| 1. Allocation of resources | Ensuring equitable distribution of medical services | facilitating access to healthcare via telesurgery; responsibility not to withhold promising new treatment | justice | yes |
| 1. Confidentiality | Confidentiality refers to the duty of healthcare professionals to restrict access to information gained from the patient. | problems related to informed consent and data protection; need for a national or international legal framework | respect for autonomy | yes |
| 1. Conflict of interest | Collision of multiple points of interest | need for a national or international legal framework | justice | yes |
| 1. Consent for the involvement of trainees in surgical procedures | Agents in the healthcare system without a graduation in their field need the consent of their treated patients. | ensuring technical expertise of surgeons | respect for autonomy | yes |
| 1. Continuous professional development | Health professionals need to update their specialised knowledge and required skills. | overreliance on new technologies; ensuring technical expertise of surgeons; responsibility not to withhold promising new treatment | beneficence | yes |
| 1. Functioning equipment and optimal operating conditions | Ensuring the best possible treatment options | facilitating access to healthcare via tele-surgery; overreliance on new technologies; ensuring technical expertise of surgeons; responsibility not to withhold promising new treatment | beneficence | yes |
| 1. Good communication skills | Healthcare professionals need to interact appropriately with patients and should be able to explain all relevant information to lay people. | problems related to informed consent and data protection | respect for autonomy | yes |
| 1. Informed consent for surgery | Informed consent of patients refers to their ability to receive sufficient information to autonomously decide on medical interventions. | problems related to informed consent and data protection; overreliance on new technologies | respect for autonomy | yes |
| 1. Legal and regulatory issues | Aspects of medical interventions which can play a role in a legal framework | need for a national or international legal framework | justice | yes |
| 1. Minimizing harm (including pain control) | Optimizing the endurability of medical interventions | responsibility not to withhold promising new treatment; facilitating access to healthcare via tele-surgery; ensuring technical expertise of surgeons | non-maleficence | yes |
| 1. Professionalism | Healthcare professionals must comply with the standards applicable to their area of practice. | ensuring technical expertise of surgeons; overreliance on new technologies; need for a national or international legal framework; problems related to informed consent and data protection | beneficence | yes |
| 1. Recognizing the limits of one’s professional competence | Awareness of the individual capabilities in providing healthcare services | overreliance on new technologies; ensuring technical expertise of surgeons | non-maleficence | yes |
| 1. Research and auditing | Re-evaluation of medical interventions according to the state of research | responsibility not to withhold promising new treatment; facilitating access to healthcare via tele-surgery; ensuring technical expertise of surgeons | non-maleficence | yes |
| 1. Research and innovation in surgery | Progresses in the field of surgical knowledge and practice. | overreliance on new technologies; ensuring technical expertise of surgeons; responsibility not to withhold promising new treatment | non-maleficence | yes |
| 1. Responsible conduct | Awareness of and concern for the consequences of medical interventions. | overreliance on new technologies; ensuring technical expertise of surgeons; responsibility not to withhold promising new treatment | beneficence | yes |
| 1. Surgical competence | Surgical competence refers to the ability to appropriately assess and treat all conditions of the human body that require surgical intervention. | ensuring technical expertise of surgeons; facilitating access to healthcare via tele-surgery | non-maleficence | yes |
| 1. Truth-telling (to patients, relatives, and colleagues) | Truth telling refers to the physician’s duty not to withhold vital information regarding any medical interventions. | problems related to informed consent and data protection; overreliance on new technologies; ensuring technical expertise of surgeons; responsibility not to withhold promising new treatment; need for a national or international legal framework | respect for autonomy | yes |
| 1. Disclosure and discussion of surgical complications including medical errors | Offering transparency and sufficient information about possible risks of medical interventions | problems related to informed consent and data protection | non-maleficence | no |
| 1. Respecting human rights | Respect for the principles of the International Bill of Human Rights | facilitating access to healthcare via tele-surgery; problems related to informed consent and data protection; need for a national or international legal framework | justice | no |
| 1. Respecting patient’s requests (for procedures/particular surgeons) | The patient's preferences regarding their treatment should play a central role in medical decision-making as far as possible. | facilitating access to healthcare via tele-surgery | respect for autonomy | no |
| 1. Shared decision making | Medical decision-making must take into account patients' preferences regarding their treatment and support them in choosing the most appropriate options together with healthcare professionals. | problems related to informed consent and data protection | respect for autonomy | no |
| 1. Whistle blowing | Public disclosure of confidential information | need for a national or international legal framework; problems related to informed consent and data protection | justice | no |

1. Ethical issues have been extracted from prevalent ethical frameworks for surgical ethics deal with virtue ethics or casuistry approaches [28], a principled approach for surgical complications [30], a principled approach for surgical research [31], and ethical challenges of innovation in surgery [27, 32]. [↑](#footnote-ref-1)
2. Principles of biomedical ethics are respect for autonomy, beneficence, non-maleficence, and justice according to Beauchamp and Childress [30]. [↑](#footnote-ref-2)
