# Supplementary material for: Intraoperative application of mixed and augmented reality for digital surgery: a systematic review of ethical issues
Source: Front Surg. 2024 Mar 14;11:1287218. doi: 10.3389/fsurg.2024.1287218 (PMC10972963; doi:10.3389/fsurg.2024.1287218)
Supplement: Supplementary file 2 [file Datasheet2.docx]

**Supplement 2.** Included Publications.

| **Nr** | **Title** | **First author** | **Country** | **Year** | **Journal** | **Article Type** |
| --- | --- | --- | --- | --- | --- | --- |
| 1 | Tracking Accuracy of a Stereo Camera-Based Augmented Reality Navigation System for Orthognathic Surgery | Jaemyung Ahn | Korea | 2019 | Journal of Oral and Maxillofacial Surgery | n/a |
| 2 | A Delphi consensus statement for digital surgery | Kyle Lam | Various | 2022 | NPJ Digital Medicine | Original Article |
| 3 | Defining digital surgery for the future | Marium M. Raza | USA | 2022 | NPJ Digital Medicine | Editorial |
| 4 | The impact of extended reality on surgery: a scoping review | James Zhang, Victor Lu | UK | 2023 | International Orthopaedics | Review |
| 5 | Mixed reality in oral and maxillofacial surgery: a symbiosis of virtual and augmented reality or a pointless technological gadget? | Bartella, AK | Germany | 2021 | International journal of computerized dentistry | Application |
| 6 | Augmented reality in craniomaxillofacial surgery: added value and proposed recommendations through a systematic review of the literature | M. Benmahdjoub | Netherlands | 2020 | Journal of Oral and Maxillofacial Surgery | Review |
| 7 | The Use of Augmented Reality to Guide the Intraoperative Frozen Section During Robot-assisted Radical Prostatectomy | Lorenzo Bianchi | Italy | 2021 | European Urology | n/a |
| 8 | Microscope-Based Augmented Reality in Degenerative Spine Surgery: Initial Experience | Barbara Carl | Germany | 2019 | World Neurosurgery | Original Article |
| 9 | Augmented Reality in Transsphenoidal Surgery | Barbara Carl | Germany | 2019 | World Neurosurgery | Original Article |
| 10 | Discussion: Mixed Reality with HoloLens: Where Virtual Reality Meets Augmented Reality in the Operating Room | Austin D. Chen | USA | 2017 | Plastic and Reconstructive Surgery | Discussion |
| 11 | Enhancing Reality: A Systematic Review of Augmented Reality in Neuronavigation and Education | James Cho | USA | 2020 | World Neurosurgery | Review |
| 12 | Mixed reality for visualization of orthopedic surgical anatomy | Dimitrios Chytas | Greece | 2018 | World Journal of Orthopaedics | Editorial |
| 13 | Comment on "Intraoperative 3D Hologram Support With Mixed Reality Techniques in Liver Surgery" | Dimitrios Chytas | Greece | 2021 | Annals of Surgery | Commentary |
| 14 | Display-Technologien für Augmented Reality in der Medizin | Ulrich Eck | Germany | 2018 | Der Unfallchirurg | Originial Article |
| 15 | Augmented reality in open surgery | Benish Fida | Italy | 2018 | Updates in Surgery | Review |
| 16 | Augmented Reality (AR) in Orthopedics: Current Applications and Future Directions | Andrew A. Furman | USA | 2021 | Current Reviews in Musculoskeletal Medicine | n/a |
| 17 | A feasibility study of a new method to enhance the augmented reality navigation effect in mandibular angle split osteotomy | Yuan Gao | China | 2019 | Journal of Cranio-Maxillo-Facial Surgery | n/a |
| 18 | The Use of Augmented Reality Navigation to Optimize the Surgical Management of Craniofacial Fibrous Dysplasia | Yuan Gao | China | 2021 | Journal of Oral and Maxillofacial Surgery | n/a |
| 19 | Testing Smart Glasses in urology: Clinical and surgical potential applications | E. García-Cruz | Spain | 2018 | Actas Urologicas Espanolas | n/a |
| 20 | CORR Insights®: Can Augmented Reality Be Helpful in Pelvic Bone Cancer Surgery? An In Vitro Study | Craig Gerrand | UK | 2018 | Clinical Orthopaedics and Related Research | Insights |
| 21 | Percutaneous computed tomography-guided lung biopsies: preliminary results using an augmented reality navigation system | Rosario Francesco Grasso | Italy | 2012 | Tumori | n/a |
| 22 | Augmented Reality for Head and Neck Carcinoma Imaging: Description and Feasibility of an Instant Calibration, Markerless Approach | Christina Gsaxner | Austria | 2020 | Computer Methods and Programs in Biomedicine | n/a |
| 23 | Clinical Feasibility of a Wearable Mixed-Reality Device in Neurosurgery | Fatih Incekara | Netherlands | 2018 | World Neurosurgery | Original Article |
| 24 | Telemedicine, Telementoring, and Telesurgery for Surgical Practices | Man Li Jin | USA | 2021 | Current Problems in Surgery | n/a |
| 25 | Virtual and Augmented Reality in Cardiovascular Care | Christian Jung | Various | 2021 | Cardiovascular Imaging | State-Of-The-Art Paper |
| 26 | Making Augmented and Virtual Reality Work for the Plastic Surgeon | Jonathan Kanevsky | Canada | 2019 | Annals of Plastic Surgery | Texts and Context |
| 27 | Intraoperative holography navigation using a mixed-reality wearable computer during laparoscopic cholecystectomy | Michiko Kitagawa | Japan | 2021 | Surgery | n/a |
| 28 | The Ethical Digital Surgeon | Kyle Lam | UK | 2021 | Journal of Medical Internet Research | Viewpoint |
| 29 | Virtual and Augmented Reality in Liver Surgery | Hauke Lang | Germany | 2019 | Annals of Surgery | Commentary |
| 30 | Applications of Head-Mounted Displays and Smart Glasses in Vascular Surgery | Fabien Lareyre | France | 2021 | Annals of Vascular Surgery | Review |
| 31 | What Is Your Reality? Virtual, Augmented, and Mixed Reality in Plastic Surgery Training, Education, and Practice | Gordon K. Lee | Spain | 2020 | Plastic and Reconstructive Surgery | Special Topic |
| 32 | Virtual Reality and Augmented Reality—Translating Surgical Training into Surgical Technique | R. Randall McKnight | USA | 2020 | Current Reviews in Musculoskeletal Medicine | n/a |
| 33 | Feasibility of the Application of Holographic Augmented Reality in Endovascular Surgery Using Microsoft HoloLens Head-Mounted Display | Claude Mialhe | France | 2021 | Annals of Vascular Surgery | Selected technique |
| 34 | Applications of Extended Reality in Ophthalmology: Systematic Review | Chee Wui Ong | Singapore | 2021 | Journal of Medical Internet Research | Review |
| 35 | Three-dimensional Elastic Augmented-reality Robot-assisted Radical Prostatectomy Using Hyperaccuracy Three-dimensional Reconstruction Technology: A Step Further in the Identification of Capsular Involvement | Francesco Porpiglia | Italy | 2019 | European Urology | n/a |
| 36 | Three-dimensional Augmented Reality Robot-assisted Partial Nephrectomy in Case of Complex Tumours (PADUA 10): A New Intraoperative Tool Overcoming the Ultrasound Guidance | Francesco Porpiglia | Italy | 2020 | European Urology | n/a |
| 37 | Through the HoloLens™ looking glass: augmented reality for extremity reconstruction surgery using 3D vascular models with perforating vessels | Philip Pratt | UK | 2018 | European Radiology Experimental | Technical Note |
| 38 | Response to Comment on ‘‘Mixed Reality in Visceral Surgery: Development of a Suitable Workflow and Evaluation of Intraoperative Usecases’’ | Moritz Queisner | Germany | 2019 | Annals of Surgery | Correspondance |
| 39 | Mixed reality applications in urology: Requirements and future potential | Gerd Reis | Germany | 2021 | Annals of Medicine and Surgery | Review |
| 40 | Current and Future Applications of Virtual, Augmented, and Mixed Reality in Cardiothoracic Surgery | Amir H. Sadeghi | Netherlands | 2020 | Annals of Thoracic Surgery | n/a |
| 41 | Augmented, virtual and mixed reality in spinal surgery: A real-world experience | Daisuke Sakai | Japan | 2020 | Journal of Orthopaedic Surgery | Review |
| 42 | Grand Adventure of Augmented Reality in Landscape of Surgery | Fatemeh Salehahmadi | Iran | 2019 | World Journal of Plastic Surgery | Review |
| 43 | Comment on: “A Novel Evaluation Model for a Mixed-Reality Surgical Navigation System: Where Microsoft HoloLens Meets the Operating Room” | Marios Salmas | Cyprus | 2020 | Surgical Innovation | Correspondance |
| 44 | Evaluation of augmented-reality based navigation for brain tumor Surgery | Makoto Satoh | Japan | 2021 | Journal of Clinical Neuroscience | n/a |
| 45 | The New Frontier: A Review of Augmented Reality and Virtual Reality in Plastic Surgery | Lohrasb R. Sayadi | USA | 2019 | Aesthetic Surgery Journal | Originial Article |
| 46 | Augmented reality technology for preoperative planning and intraoperative navigation during hepatobiliary surgery: a review of current methods | Rui Tang | China | 2018 | Hepatobiliary & Pancreatic Diseases International | Review |
| 47 | Virtual and augmented reality for biomedical applications | Mythreye Venkatesan | USA | 2021 | Cell Reports Medicine | Review |
| 48 | Virtual, augmented, and mixed reality applications in orthopedic surgery | Jens T. Verhey | USA | 2019 | Int J Med Robotics Comput Assist Surg | Review |
| 49 | 3D mixed‑reality visualization of medical imaging data as a supporting tool for innovative, minimally invasive surgery for gastrointestinal tumors and systemic treatment as a new path in personalized treatment of advanced cancer diseases | Ryszard Wierzbick | Poland | 2021 | Journal of Cancer Research and Clinical Oncology | Original Article |
| 50 | Does intraoperative navigation improve the accuracy of mandibular angle osteotomy: Comparison between augmented reality navigation, individualised templates and free-hand techniques | Ming Zhu | China | 2018 | Journal of Plastic, Reconstructive & Aesthetic Surgery | n/a |
