# Supplementary material for: Intraoperative application of mixed and augmented reality for digital surgery: a systematic review of ethical issues
Source: Front Surg. 2024 Mar 14;11:1287218. doi: 10.3389/fsurg.2024.1287218 (PMC10972963; doi:10.3389/fsurg.2024.1287218)
Supplement: Supplementary file 3 [file Datasheet3.docx]

**Supplement 3.** Codebook with code system.

| **Patient-Physician Relationship**  **Definition**  The patient-physician relationship as a service is subject to processes of change.  **Coding rules**  trust, truth-telling, patient perspective, financial aspects  **Anchor examples**  Additionally, in telementored surgical cases, yet to be clarified is the financial relationship between the patient and the telementoring physician. (*Jin-2021-Telemedicine, telementoring, and tele.pdf, S. 46: 745*)  In the present study, the patient was not exposed to the 3D dataset due to a potential psychologic burden. Even for nonmedical professionals, the perception of proximity to intracranial structures is strikingly visible, and the patient was already expressing signs of distress. (*Bartella-2021-Mixed reality in oral and maxillofacial surgery, S. 74*)  Preservation of privacy and confidentiality is essential, not only to safeguard patient autonomy but also to ensure patient trust (Lam-2022-A Delphi consensus statement for digi.pdf, S. 3: 2148)  Ethical aspects are also a major consideration when investigating new devices for surgical practice. Such technology not only raises concerns for data privacy and protection but should also guarantee accuracy, safety and security for a potential use in patients. According to the European Union’s General Data Protection Regulation (GDPR), the patient’s consent is a main concern for the use HMD or smart glasses, as the device processes medical information. Besides, the device needs to be configured to protect the patients’ data.40 (Lareyre-2021-Applications of Head-Mounted Disp.pdf, S. 14: 4519)  It is unclear how patients undergoing surgery in the future operating room will be protected from data misuse or how hospital providers and patients will be reimbursed by the companies that use them to create their digital products. (Lam-2021-The Ethical Digital Surgeon.pdf, S. 1: 3156) |  |
| --- | --- |
| Confidentiality |  |
| Ensuring |  |
| ... privacy | 12 |
| ... cybersecurity | 11 |
| ... patient data is not being sold to companies without consent | 1 |
| ... only entitled users are granted access to patient data | 2 |
| Awareness of |  |
| ... the patient's perspective towards MR/AR | 4 |
| ... reimbursing patients for using their data | 2 |
| Ensuring |  |
| ... truth-telling | 1 |
| ... trust within patient-physician relationship | 1 |
|  |  |
| **Informed consent**  **Definition**  Informed consent of patients refers to their ability to receive sufficient information to autonomously decide on medical interventions.  **Coding rules**  Information disclosure, opt-out option  **Anchor example**  Appropriate consent must be sought from patients to ensure patient autonomy and privacy. (*Lam-2022-A Delphi consensus statement for digi.pdf, S. 3: 4015*)  As digital surgery is rapidly incorporated into clinical practice, it is also essential that we are able to explain digital surgery to patients clearly and consistently, especially in the context of data collection and processing for digital surgery applications.  *(Lam-2022-A Delphi consensus statement for digi.pdf, S. 2: 1251)* |  |
| Information |  |
| Ability to explain digital surgery to patients | 2 |
| Obtaining consent for |  |
| ... risks of MR/AR technologies | 4 |
| ... involving trainees in surgery | 4 |
| ... processing of patient data | 12 |
| ... telesurgery | 1 |
| **Professionalism**  **Definition**  Healthcare professionals must comply with the standards applicable to their area of practice.  **Coding rules**  continuous professional development, learning curve, surgical competence  **Anchor examples**  “Binary surgeons” who do not have access to digital robotic platforms or who choose to reject them will be left in a perilous position where their performance will be compared with digitally augmented colleagues regardless of whether they “opt out.” (*Lam-2021-The Ethical Digital Surgeon.pdf, S. 2: 799*)  There is a steep learning curve in integrating this technology for new adopters, especially for those with limited immersion in AR, VR or MR environments. This carries a potentially high cost in training personnel with few technologies on the market holding an oligopoly. (*Sakai-2020-Augmented, virtual and mixed realit.pdf, S. 7: 4314*)  Second, the learning curve of heads-up surgery has yet to be studied comprehensively. (*Ong-2021-Applications of Extended Reality in O.pdf, S. 10: 3293*) |  |
| Continuous professional development |  |
| Opportunity |  |
| ... of access to experts world-wide (telementoring) | 6 |
| ... to avoid negative training (inappropriate behaviors) | 2 |
| Ensuring |  |
| ... legal literacy | 1 |
| ... AI literacy | 10 |
| ... data literacy | 3 |
| ... technical literacy | 3 |
| ... improvement of the learning curve | 27 |
| Surgical competence |  |
| No standard of certifying digital surgery training | 1 |
| Inexperienced surgeons use MR/AR | 3 |
| Ensuring thorough operation planning | 3 |
| Recognizing the limits of one’s professional competence | 2 |
| MR/AR enables new maneuvers for surgeons | 34 |
| **Research and Innovation**  **Definition**  Progresses in the field of surgical knowledge and practice.  **Coding rules**  research ethics, further research needed  **Anchor examples**  Experimenting on larger numbers of subjects, trials and operators to obtain statistically significant and representative results. (*Benmahdjoub-2020-Augmented reality in cranioma.pdf, S. 8*) |  |
| Need for |  |
| ... continuous research and auditing | 40 |
| ... comparative studies evaluating clinical endpoints | 5 |
| ... active participation in developing MR/AR to be successful | 2 |
| ... evaluation studies through user assessment | 1 |
| ... definition of the new gold standard (2D vs. 3D) | 1 |
| ... cost-effectiveness studies | 1 |
| Innovation anxiety |  |
| Mitigating resistance against innovation | 3 |
| Recognizing the fact that MR/AR is a technology under development | 4 |
| Research ethics |  |
| Demand for more and larger RCTs before implementation | 2 |
| Opt-out option for patients regarding use of their data | 1 |
| Evidence must demonstrate clear advantages | 3 |
| Debated ethicality of control groups in surgical research | 2 |
| **Legal and Regulatory Issues**  **Definition**  Aspects of medical interventions that can play a role in legal or regulatory frameworks.  **Coding rules**  litigation, responsibility, accountability, regulation, standardization  **Anchor examples**  Although digital surgeons will probably remain accountable for the decisions that they make, it is now possible that they will also have to contend with automation bias, opaque algorithms, and a rapidly evolving ecosystem of sophisticated cloud-based platforms and connected hardware. This will in turn create new challenges for consent and litigation, which are as of yet untested. (*Lam-2021-The Ethical Digital Surgeon.pdf, S. 2: 166*) |  |
| Data Protection |  |
| Issues of data protection and data ownership | 10 |
| Liability |  |
| Unclear liability of surgeons who do not follow decision support | 3 |
| Surveilled surgeons fear litigation in medical negligence cases | 5 |
| Standard Procedures |  |
| Need for regulatory framework for clinical trainees | 2 |
| Lack of standardization | 4 |
| No standard operating procedures for proper patient consent | 4 |
| No standard for informed consent | 5 |
| **Functioning equipment and optimal operating conditions**  **Definition**  Ensuring the best possible treatment options  **Coding rules**  comfort, ergonomics, focus shift, field of view, setup time, clinical workflow implementation, cybersickness  *In our coding, we have only taken text passages into account if ethical conflicts arise from them. We did not take into account all various technical advantages of AR-based technologies, if they could not be added for the code "not withholding better treatment"*  **Anchor examples**  Another concern is related to the ergonomics and the comfort of the users. HMD devices are still heavy and the weight varies between 500 g to 645 g depending on the device and manufacturer. Smart glasses weigh between 46 g to 140 g. This can cause discomfort and fatigue to the user, especially for long surgical procedures. HMD are adjusted to head size and are compatible with eye glasses. Several users also report cybersickness such as nausea, visual discomfort, dizziness, headaches, eye strains or dry eyes. An adjustment period and an appropriate training is also required for the operators to get familiar and use the device efficiently. (*Lareyre-2021-Applications of Head-Mounted Disp.pdf, S. 14: 3858*) | 2 |
| Ensuring |  |
| ... improvement of comfort, ergonomics, and usability of device | 37 |
| ... awareness of the limitations of MR/AR technology | 15 |
| ... evidence-based implementation | 1 |
| ... hygienic requirements like sterile equipment | 10 |
| ... accuracy of super-imposed images | 6 |
| ... surgeon's motion, peripheral vision, and general perception | 7 |
| … the ability to toggle the HMD on and off to avoid distraction | 6 |
| Mitigating the risk of |  |
| ... registration errors |  |
| ... due to auto-registration | 2 |
| ... due to re-registration | 5 |
| ... due to deformation of soft tissues | 6 |
| ... due to position shifts | 5 |
| ... due to inadvertently touching the reference frame | 1 |
| ... segmentation errors | 2 |
| ... tracking errors | 3 |
| ... obscuration of the operating field | 7 |
| ... impaired accuracy | 10 |
| ... delayed reaction time of the equipment (latency) | 7 |
| ... decreased usability | 2 |
| ... attention shift and dissociation | 13 |
| ... inattentional blindness | 3 |
| ... decreased acceptability due to laborious adjustments | 2 |
| Need for maintaining redundant standard procedures (dead loss) |  |
| ... due to patient safety protocols | 1 |
| ... due to loss of internet connection | 1 |
| ... due to dead loss ("blue screen") | 2 |
| ... due to memory issues | 1 |
| ... due to battery issues | 5 |
| Mitigating health risks for users of MR/AR technology like | 1 |
| ... cybersickness, motion sickness, vertigo, and nausea | 13 |
| ... headache | 6 |
| ... ophthalmic syndromes | 5 |
| ... discomfort | 4 |
| **Allocation of resources**  **Definition**  Ensuring equitable distribution of medical services by access to healthcare.  **Coding rules**  access to healthcare, cost-effectiveness  **Anchor example**  The introduction of additional technology in the operating room could further increase the cost of operating times and procedures. For many procedures, the benefit of AR or VR may not be outweighed by the added cost of integrating these technologies into the clinical setting. As mixed reality devices come to market, evaluation of the benefit to the patient must be compared with the increased costs extensively.  Hupp et al describe the skepticism that clinicians and patients must have on the increasing cost of technology to the marginal returns in the form of positive outcomes. The article examines how while the technology involved in implantology is evolving, so are the associated costs, yet patient outcomes have not changed drastically enough to merit the tremendous increase in cost to the patient. (*Kanevsky-2019-Making Augmented and Virtual Rea.pdf, S. 5: 1272*) |  |
| Reduced costs |  |
| ... due to higher number of procedures executed in the same time | 9 |
| ... through in-house development compared to imported devices | 1 |
| Increased costs |  |
| ... while little increase in benefits | 13 |
| ... due to expensive equipment | 9 |
| ... due to high set-up costs | 8 |
| ... due to presence of additional personnel | 3 |
| ... due to 3D model work | 2 |
| ... due to expensive training personnel | 2 |
| **Minimizing harm**  **Definition**  Optimizing the endurability of medical interventions.  **Coding rules**  safety, radiation exposure, risks, reducing procedure time  **Anchor example**  Successful implementation of new technologies requires expertise in the problems and limitations of surgical practice, a deep understanding of which new tools may help surgeons, close interaction between clinicians, scientists, and industry, an ethical framework that ensures the safe application of new technologies and protects the rights of individuals and their personal data, and a stepwise evidence-based approach to implementation. Therefore, if patients are to benefit from AR technologies, new studies are required that prove their benefit over existing techniques, for example, by demonstrating better resection margins and benefits for patients in terms of morbidity and physical functioning. (*Gerrand-2018-CORR Insights®_ Can Augmented Rea.pdf, S. 2: 1823*) | 2 |
| Reducing |  |
| ... invasiveness | 2 |
| ... radiation exposure | 11 |
| ... procedure time | 9 |
| ... task load and cognitive load of surgeons | 3 |
| Awareness |  |
| ... when critical structures are threatened | 3 |
| ... of high-risk clinical intervention | 3 |
| ... of possible errors | 10 |
| Ensuring |  |
| ... redundant conventional techniques as safeguardings | 2 |
| ... better outcomes through research | 21 |
| **Good communication skills of surgeons**  **Definition**  Healthcare professionals need to interact appropriately with colleagues and patients and should be able to explain all relevant information to lay people.  **Coding rules**  patient-centered, patient-physician-communication  **Anchor example**  Panellists agreed that there is a lack of framework or experience within the majority of institutions for the setting up of fair partnerships between healthcare and commercial entities. They highlighted issues surrounding inequality of power and differing motives between hospitals and commercial companies. Finally, panellists agreed that commercial partnerships may result in restriction on the ability of hospitals to report results.  *(Lam-2022-A Delphi consensus statement for digi.pdf, S. 4: 4210)*  We strongly support these ethical considerations and think that AR/VR should always enhance the real-world patient–provider relationship. Digital applications must not distract us from the patient: Physicians should participate in the progress and evolution of these virtual and digital advances to ensure patient-centered developments.  (*Jung-2021-Virtual and Augmented Reality in Car.pdf, S. 11: 2913*) |  |
| ... with patients | 4 |
| ... with colleagues | 7 |
| ... with technicians | 5 |
| ... with companies | 9 |
| **Ability to exercise sound judgment**  **Definition**  Healthcare professionals must be able to assess all disease-relevant conditions of the human body and select the appropriate medical interventions on this basis.  **Coding rules**  selection of appropriate treatment method, personalization  **Anchor examples**  Recently, Kellmeyer et al proposed 3 priorities: First, human-human should be preferred over human-machine interactions; second, critical human values, including dignity and autonomy, should be at the center of VR technology; and third, VR systems should be patient centered and not for professional customers. We strongly support these ethical considerations and think that AR/VR should always enhance the real-world patient–provider relationship. Digital applications must not distract us from the patient: Physicians should participate in the progress and evolution of these virtual and digital advances to ensure patient-centered developments. (*Jung-2021-Virtual and Augmented Reality in Car.pdf, S. 11: 2592*)  The ultimate goal of intraoperative implementation of XR technology in the field of cardiothoracic surgery must benefit the surgeon and patient and improve quality of care and health outcomes. (*Sadeghi-2020-Current and Future Applications o.pdf, S. 5: 6195*) |  |
| Decision-making |  |
| More certainty in decision-making | 5 |
| Avoidance of overreliance on new technology and false safety | 18 |
| Awareness of possible |  |
| ... bias towards MR/AR hyped by marketing departments | 2 |
| ... ethical issues | 7 |
| ... complications like position shifts | 9 |
| Selection of the appropriate |  |
| ... application area | 16 |
| ... device for the intended purpose | 5 |
| ... technology (AR, MR or VR) | 8 |
| Not withholding MR/AR if it performs better | 29 |
|  |  |
